# Predicting dropout in outpatient dialectical behavior therapy with patients with borderline personality disorder receiving psychiatric disability

**DOI:** 10.1186/s40479-016-0043-3

**Published:** 2016-09-01

**Authors:** Sara J. Landes, Samantha A. Chalker, Katherine Anne Comtois

**Affiliations:** 1 Department of Psychiatry and Behavioral Sciences, University of Washington at Harborview Medical Center, Box 359911, 325 Ninth Avenue, Seattle, WA 98104 USA; 2 Department of Psychiatry, Division of Health Services Research, University of Arkansas for Medical Sciences, 4301 W. Markham St., #755, Little Rock, AR 72205 USA; 3 VISN 16 Mental Illness Research, Education, and Clinical Center (MIRECC), Central Arkansas Veterans Healthcare System, 2200 Fort Roots Drive, North Little Rock, 72114 AR USA; 4 Department of Psychology, The Catholic University of America, O’Boyle Hall, Room 339, 620 Michigan Ave. NE, Washington, DC, 20064 USA

**Keywords:** Dropout, Dialectical behavior therapy, Predict, Borderline personality disorder, Skills group

## Abstract

**Background:**

Rates of treatment dropout in outpatient Dialectical Behavior Therapy (DBT) in the community can be as high as 24 % to 58 %, making dropout a great concern. The primary purpose of this article was to examine predictors of dropout from DBT in a community mental health setting.

**Methods:**

Participants were 56 consumers with borderline personality disorder (BPD) who were psychiatrically disabled participating in a larger feasibility trial of Dialectical Behavior Therapy- Accepting the Challenges of Exiting the System. The following variables were examined to see whether they predicted dropout in DBT: age, education level, baseline level of distress, baseline level of non-acceptance of emotional responses, and skills module in which a consumer started DBT skills group. These variables were chosen based on known predictors of dropout in consumers with BPD and in DBT, as well as an interest in what naturally occurring variables might impact dropout.

**Results:**

The dropout rate in this sample was 51.8 %. Results of the logistic regression show that younger age, higher levels of baseline distress, and a higher level of baseline non-acceptance of emotional responses were significantly associated with dropout. The DBT skills module in which an individual started group did not predict dropout.

**Conclusions:**

The implications of these findings are that knowledge of consumer age and pretreatment levels of distress and non-acceptance of emotional responses can impact providers’ choice of commitment and treatment strategies to reduce dropout. Future research should examine these strategies, as well as the impact of predictor variables on outcome and reasons for dropout.

## Background

Dialectical behavior therapy (DBT [1]) is an evidence-based psychotherapy developed for suicidal individuals with borderline personality disorder (BPD). In an outpatient setting, DBT includes group skills training, individual therapy, phone coaching, and therapist consultation team. DBT implemented with all of these components is called standard DBT and modal completion takes one year. There have been at least 11 randomized controlled trials (RCTs) on DBT and DBT has been shown to be helpful at reducing suicidal behavior, non-suicidal self-injury, depression, hopelessness, anger, substance dependence, symptoms of eating disorders, and improving psychosocial adjustment and treatment retention in studies primarily with consumers with BPD (as described in Landes and Linehan [2]). DBT’s effectiveness has also been demonstrated in “real-world” settings (e.g., [3, 4]).

### Dropout in consumers with BPD and in DBT

In studies of treatment dropout with consumers with BPD, predictors of dropout include anger [5–7], high levels of hostility [7], impulsiveness [5, 6], higher trait anxiety [5], higher baseline experiential avoidance [5], and history of a least one suicide attempt [8]. Similar to the broader psychotherapy dropout literature, younger age [9–14] and lack of motivation for change [6, 7, 11, 13, 15–18] are also predictors of dropout in consumers with BPD. However, there are some conflicting findings. In a meta-analysis of dropout in consumers with BPD, Barnicot et al. [5] found that none of the examined socio-demographic variables predicted dropout, including age, gender, marital status, living alone, education, employment status, race, or religion. This is dissimilar to consistent predictors of dropout in the broader psychotherapy dropout literature, where less education is a predictor [9, 10, 14, 19–21]. Lengths of hospitalizations, severity of BPD symptoms, and comorbid diagnoses were also not predictive of dropout [5].

In DBT, a specific treatment for consumers with BPD, treatment dropout is defined as missing four consecutive appointments of any one treatment component (e.g., missing four groups in a row, missing four individual appointments in a row) [1]. DBT dropout rates have ranged from 17 % to 39 % in research studies [22–28].

DBT in the community tends to have higher dropout rates, ranging from 24 % to 58 % [4, 29–31]. The higher dropout rate of 52 % in Priebe et al. [4] may be due to a stricter definition of dropout. In this study, dropout was defined as missing four consecutive sessions of skills group, individual sessions, *or any combination of the two*. Therefore, the dropout rate would have to be higher, given that a consumer who missed two weeks of treatment (both individual and skills group) would be considered a dropout in this study, but not in other DBT programs. Feigenbaum and colleagues (2012) discuss their high dropout rate of 58 % and posit inclusion of clients with anti-social and paranoid personality disorders as one possible reason, as clients with either or both of these diagnoses accounted for 53 % of dropouts. They also note that the consultation team determined that one therapist who left the team had been providing poor quality DBT; all of that therapist’s clients subsequently dropped out after the therapist left, accounting for 36 % of those who dropped out.

Few studies have examined predictors of dropout in DBT. The majority examined dropout in either inpatient or shortened forms of DBT. All of the studies examining standard outpatient DBT defined dropout more strictly than the original Linehan text [1].

In inpatient DBT, dropout rates have ranged from 10 % to 46 %. In two studies, no significant differences were found between treatment completers and dropouts [32, 33]. Others have found that fewer lifetime suicide attempts, higher experiential avoidance, substance use disorder, younger age, antisocial personality disorder, and more than 86 weeks in a psychiatric hospital predicted dropout [7, 34, 35]. In a brief intensive outpatient DBT program, Perroud et al. [20] found that only low educational level predicted dropout. Soler et al. [17] examined a three-month group DBT treatment and lack motivation for change was the only predictor of dropout.

Gaglia et al. [36] examined dropout from standard outpatient DBT in the United Kingdom National Health Service. They defined dropout more strictly, as did Priebe et al. [4]. Presence of care coordination history was the only predictor of dropout. Care coordination involves having a key worker who monitors symptoms and coordinates care, which includes meeting regularly. White and colleagues^1^ (unpublished observations) examined predictors of completion of standard DBT in an outpatient private practice setting and defined dropout more strictly (missing three consecutive of either skills group or individual sessions). Younger age, more pretreatment sessions, and higher levels of baseline psychological distress predicted dropout.

In considering factors that may predict dropout, another area of interest is the variation in treatment that naturally occurs as a result of an ongoing program or that providers have the ability to control, such as consumers starting the skills groups at different times and beginning treatment in different skill modules. In the skills training manual, Linehan [37] states there is no empirical data to support a specific order and that because mindfulness skills are woven throughout each module, mindfulness is first. The suggested order of remaining modules is interpersonal effectiveness, emotion regulation, and distress tolerance.

### Current study

Given the existing literature and lack of available studies examining dropout as defined in Linehan [1] in a community outpatient setting, the current study examined the following variables to see whether they predicted dropout in DBT: age, education level, baseline level of distress, baseline level of non-acceptance of emotional responses (as a proxy for experiential avoidance), and skills module in which a consumer starts skills group. These variables were chosen based on known predictors of dropout in consumers with BPD and in DBT, as well as an interest in what naturally occurring variables might impact dropout. Dropout was defined as in Linehan [1], as missing four consecutive appointments of any one treatment component. The hypotheses are that the following variables will predict dropout: 1) younger age, 2) lower level of education, 3) higher level of baseline distress, and 4) higher level of baseline non-acceptance of emotional responses. A final exploratory hypothesis is that 5) the skills module in which a consumer starts skills group will predict dropout.

## Method

### Participants

Participants in the larger study were 63 consumers with BPD who were psychiatrically disabled participating in a feasibility trial of Dialectical Behavior Therapy- Accepting the Challenges of Exiting the System (DBT-ACES) [38]. DBT-ACES involves one year of standard DBT, followed by a second year of modified DBT focused on obtaining living wage employment and decreased reliance on the mental health system. Inclusion criteria were receiving government psychiatric disability, meeting a county “exceptional care” criteria indicating severe psychopathology and risk of hospitalization, meeting criteria for BPD, being between 18 and 60 years of age, consenting to treatment with a focus on employment and self-sufficiency, and receiving pharmacotherapy within the study. Those with a suspected IQ of less than 70, life threatening anorexia, impending jail or prison for more than three weeks, a court order to treatment, insufficient proficiency in the English language, or current substance dependence with use in the past 90 days were excluded.

For the current study, 56 participants from the larger study were included in the analyses. The remaining seven participants were excluded because data were missing for variables of interest. Participants ranged in age from 19 to 58 years (*M* = 36.77, *SD* = 10.56). The majority was women (75.4 %) and identified as Caucasian (77 %). The majority (96 %) met criteria for an Axis I disorder, including depressive disorders (46 %), bipolar disorder (25 %), and anxiety disorders (25 %). See Table 1 for participant demographics.

**Table 1:** Participant demographics

Variable	*N*	Valid %
Gender		
Female	46	75.4
Male	15	24.6
Transgender	0	0
Ethnicity		
Caucasian	47	77.0
Mixed	7	11.5
Black/African American	3	4.9
Asian or Pacific Islander (includes Chinese, Japanese, Korean, Malaysian, Pakistani, Filipino, Indian, East Indian, Middle Eastern/Arab, Native Hawaiian or other Pacific Islander)	3	4.9
Latino or Latina (includes Mexican, Mexican American or Chicano, Puerto Rican, other Hispanic/Latino/Latina)	1	1.6
Education		
Less than a high school graduate	3	4.9
High school graduate or GED	15	24.6
Some college or vocational technical college	26	42.6
College graduate	8	13.1
More than a college education (includes some graduate school, Master’s degree, Professional degree)	9	14.8

### Measures/Materials

The *Structured Clinical Interview for DSM-IV, Axis I* (SCID-I) [39] and the *Structured Clinical Interview for DSM-IV, Axis II* (SCID-II) [40] were used to obtain diagnoses. Research assistants (RAs) were taught to administer the SCID. Training included watching SCID training tapes, watching at least one video of a completed interview and coding a SCID to compare ratings, coding along with a live interview, and conducting an interview with a supervisor. Diagnoses of BPD were confirmed by provider diagnosis in the medical record.

The *Peabody Picture Vocabulary Test - Revised* (PPVT-R) [41] was used to rule out mental retardation. This brief measure of verbal intelligence has the advantage of low sensitivity to learning disabilities, which are seen frequently among participants with BPD. It results in an IQ score comparable to those of other intelligence tests such as the WAIS-R and Stanford-Binet [42, 43, 44].

The *Demographic Data Schedule* (DDS; Linehan, unpublished work)^2^ was used to obtain a wide range of demographic data, including age, gender, and level of education. High concurrent validity was established by comparing DDS responses to hospital chart data for a sample of psychiatric inpatients.

The *Brief Symptom Inventory* (BSI) [44] is a 53-item self-report measure that assesses psychiatric symptoms; it was designed to be a briefer version of the SCL-90-R [45, 46]. Items describe psychiatric symptoms (e.g., “Spells of terror or panic”) and reflect 9 primary symptom dimensions: somatization, obsessive-compulsive, interpersonal sensitivity, depression, anxiety, hostility, phobic anxiety, paranoid ideation, and psychotics. Respondents rate how much they were distressed by each item during the last 4 weeks on a 5-point Likert scale ranging from 0-4 (0 = not at all and 4 = extremely). Scoring results in 3 global indices of distress: the Global Severity Index (GSI), the Positive Symptom Distress Index (PDSI), and the Positive Symptom Total (PST). The GSI is the best indicator of current level of distress and is the mean of all item ratings; higher scores indicate greater psychological distress. The 3 indices have good reliability over time [47–49] and good convergent validity with MMPI subscales [48].

The *Difficulties in Emotion Regulation Scale* (DERS) [50] is a 36-item self-report questionnaire designed to assess multiple aspects of emotion dysregulation. The range of possible scores is 36-180; higher score indicates more difficulty with emotion regulation. The measure yields a total score as well as scores on six scales derived through factor analysis: 1. Non-acceptance of emotional responses (Non-acceptance), 2. Difficulties engaging in goal directed behavior (Goals), 3. Impulse control difficulties (Impulse), 4. Lack of emotional awareness (Awareness), 5. Limited access to emotion regulation strategies (Strategies), and 6. Lack of emotional clarity (Clarity). The DERS has been found to demonstrate high internal consistency, good test-retest reliability, and adequate construct and predictive validity within an ethnically and socioeconomically diverse sample.

In the current study, Cronbach’s alpha was high for both the BDI and DERS measures; .98 and .91 respectively. For the DERS non-acceptance subscale, Cronbach’s alpha was good (.72).

#### Chart review

Chart notes from medical records were reviewed to determine the DBT skills covered in each consumer’s first skills group. In this program, standard chart notes are used and each includes the module and topic covered. Based on chart review, it was recorded whether or not the consumer started during mindfulness (yes or no) and which of the remaining modules they did first. Chart notes were used to determine whether the consumer was a treatment dropout or completer. This is routinely documented by providers.

### Procedure

All procedures were approved by the local Institutional Review Board.

#### Screening

Individuals contacted the study directly or were referred by DBT intake staff. Participants were offered one year of standard DBT followed by random assignment to DBT-ACES or a non-DBT vocational program if they completed standard DBT. Individuals who passed the phone screen were screened in person, which included the informed consent process. Participants accepted into the study completed a pre-treatment assessment.

#### Treatment & Providers

As described above, standard DBT consists of weekly individual psychotherapy, weekly DBT skills group, phone consultation, and therapist consultation team. Treatment lasts for one year. As with other ongoing DBT programs, consumers entering the program were assigned to skills groups based on availability of space and consumer’s preference for day of group. The module in which a consumer started group was not manipulated, as the larger trial was on feasibility of DBT-ACES and no modifications were made to standard DBT.

The DBT team lead was intensively trained in 1994 and has since served as a research therapist on Dr. Linehan’s treatment outcome studies and trains and supervises DBT internationally. All providers received intensive DBT training [2] or the equivalent (e.g., six month training course in a residency program) and have received supervision from the team lead or Dr. Linehan.

Treatment adherence was rated using the DBT Adherence Rating Scale; this scale generates a computed global score that ranges from 1–5. Global scores >4.0 represent DBT adherence; scores below 4.0 signal need for consultation/supervision. Tapes from individual psychotherapy sessions were randomly selected for each provider and coded by three DBT providers who have received extensive training in DBT and adherence coding. Scores for all providers ranged from 3.6 to 4.3, with an average score of 4.0 (*SD* = .17), indicating that providers were generally at adherence. Supervision and/or consultation was provided for scores lower than 4.0. These are similar to scores obtained by Linehan and colleagues [24] in a RCT of DBT.

## Data analysis

Data were analyzed using SPSS 19.0 for Windows. The effects on dropout of which skills group module consumers started in after mindfulness (i.e., interpersonal effectiveness [IE], emotion regulation [ER], vs. distress tolerance [DT]) and if they started skills group in a mindfulness module were examined using a logistic regression. Covariates included (and were entered in the following order) age, level of education, pretreatment GSI score, and pretreatment DERS non-acceptance subscale score. Because consumers were nested within providers, a general estimating equation was used to control for within provider effects [51]. The variables of age, GSI, and DERS non-acceptance were transformed into z-scores to facilitate comparison. Significance level was set at **α** = .05.

## Results

### Dropout

In this sample of consumers with BPD recruited for a study of psychiatric disability and treated in a publicly funded mental health clinic, the dropout rate was 51.8%. Table 2 describes the mean and quartiles for weeks in treatment for treatment dropouts and completers.

**Table 2:** Weeks in treatment

	Dropout	Completers
N	29	27
Mean (SD)	26.00 (16.03)	52.44 (7.10)
Quartiles		
25	10.00	51.00
50	22.00	52.00
75	41.00	56.00

### Predictors of dropout

The relationship between each variable and dropout are presented in Table 3. Findings determined by these results show that age, GSI, and DERS non-acceptance are significantly associated with dropout status; the other variables are not. With each additional standard deviation increase in age, the odds of dropout decreased by 72 %. For each standard deviation increase in GSI, the odds of dropout decreased by 78 %. Each additional standard deviation increase in DERS non-acceptance was associated with a 98 % increase in the odds of dropout. Of note, we analyzed the data using the DERS total score transformed into z-scores and had similar results. The working correlation matrix in generalized estimation equation (GEE) did not indicate any effect of provider. As dropout from DBT can vary in time throughout a year period, we considered whether time to dropout could affect the results. However, a Cox proportional hazard survival analysis predicting time to dropout with the same predictors and clustering for within provider nesting showed no difference in results and are not detailed here.

**Table 3:** Generalized estimating equation predicting dropout

Variable	OR	95% CI
Age (z-score)	0.284*	0.097-0.836
Education	1.775	0.904-3.487
GSI (z-score)	0.228*	0.140-0.369
DERS non-acceptance (z-score)	1.982*	1.372-2.862
DT module	REF	
IE module	1.299	0.368-4.587
ER module	0.568	0.125-2.579
Started in Mindfulness module	0.943	0.323-2.752

*OR* odds ratio, *CI* confidence interval *p < .05

## Discussion

The dropout rate in the current study (51.8 %) was higher than dropout rates observed in previous trials of DBT (16.7–39 %), yet was within the range of dropout observed in the community (24–58 %). This sample is different than other DBT treatment studies, as one of the inclusion criteria were that individuals had to be receiving psychiatric disability. While higher severity of impairment may lead to receiving psychiatric disability, pretreatment distress was not a predictor of dropout. It is possible that another variable related to receiving psychiatric disability may driving the dropout rate. Another possible explanation is that there were a number of individuals who may not have been a good fit for a program focused on obtaining employment or who may have entered treatment to obtain case management resources (e.g., leaving DBT after obtaining housing through case management). Additional research is needed regarding how receiving psychiatric disability and participating in a treatment focused on employment impacts treatment outcomes and dropout.

The results of the logistic regression regarding age support previous research that has shown that younger consumers [9–14] are more likely to dropout out of treatment.

The finding regarding individuals with greater levels of pretreatment distress being *less* likely to dropout of treatment is in conflict with research that has shown greater severity as being a predictor of dropout [20] and in research specific to DBT (White et al., unpublished observations). One possible explanation is that individuals with greater severity of symptoms may be a better fit for DBT and have better treatment outcomes in DBT. DBT is a complex and involved treatment designed to treat severe emotion dysregulation. Therefore, individuals with less distress who enter DBT may find that the treatment is more than they need and be more likely to dropout. This is an area for further research.

The results indicating that higher levels of baseline non-acceptance of emotional responses were a predictor of dropout are in line with previous literature demonstrating that higher baseline experiential avoidance is a predictor of dropout in consumers with BPD and/or in DBT [5, 7, 34, 35]. While non-acceptance and avoidance are different constructs, they may be related in that individuals who refuse to accept emotional responses are also likely to avoid the experience. This relationship should be examined in future research. The skills module in which an individual starts group and whether or not they started with mindfulness did not predict dropout. This is similar to the one previous study examining DBT skills module (White et al., unpublished observations).

## Conclusions & Implications

### Age, distress, and non-acceptance of emotional responses

Given that consumer age pretreatment level of distress, and pretreatment levels of non-acceptance of emotional responses are outside the control of a provider, these results may mean that these variables can inform use of treatment strategies. With this knowledge providers should be aware that younger consumers are more likely to dropout and therefore may want to put extra attention on engagement and commitment strategies with younger consumers. For example, providers may consider implementing the technology of choice of younger consumers (e.g., mobile apps, texting for phone coaching) or, if possible, have younger members together in skills groups. The findings regarding those with less psychiatric distress being more likely to dropout suggests that providers may want to consider assessing pretreatment distress with measures like the BSI and put extra attention on engagement and commitment strategies for those with lower scores – or consider whether DBT is the appropriate treatment (as it may be too much treatment). Finally, regarding consumers with higher levels of non-acceptance of emotional responses, providers may want to consider how to best orient these consumers to DBT, as one premise of DBT is that emotion dysregulation is the primary problem. Providers may want to do more assessment and exploration of the consumer’s view of the primary problem. By doing behavioral chain analysis (a functional analysis assessment tool), the provider and consumer can collaboratively see what the problem behaviors look like and if emotions and/or emotion dysregulation is related. The engagement and treatment strategies suggested here are based on the DBT model and could be assessed in further studies on dropout.

### Skill modules

The skill module in which consumers started group was not a predictor of dropout. Providers may now have evidence for not being concerned about starting consumers in modules other than interpersonal effectiveness as suggested by DBT skills training manual.

### Limitations

The primary limitation of this study is the small sample size. The characteristics of the participants also limit the generalizability of the findings. The majority of the participants were Caucasian women and all were on psychiatric disability. This limits the ability to suggest that these findings would apply to all individuals with BPD seeking therapy. In regard to the findings on skills modules, a primary limitation is that participants were not randomized to skills module. Therefore, we were not able to control for other variables that may have impacted assignment to group, such as provider preferences for certain consumers.

### Future directions

Based on the results of the current study, older consumers and those with greater levels of pretreatment distress are less likely to dropout from DBT. The skills module and whether or not consumers started in the mindfulness module did not predict dropout. Additional research is needed to replicate these results in other samples. Future research should examine whether these variables impact or predict treatment outcomes such as suicidal or self-harm behavior or symptom reduction.

Future research should also include other variables that may affect dropout, such as how satisfied consumers were during their treatment, how much their skill use increased after treatment and both their providers’ and their expectations of treatment. Examining the reasons consumers dropped out of treatment at follow up may suggest which barriers could be targeted to prevent dropout (e.g., medical problems or other quality of life changes, homelessness, transportation or other practical problems).

## Abbreviations

BPD, borderline personality disorder; BSI, brief symptom inventory; DBT, dialectical behavior therapy; DBT-ACES, dialectical behavior therapy- accepting the challenges of exiting the system; DDS, demographic data schedule; DERS, difficulties in emotion regulation scale; DT, distress tolerance; ER, emotion regulation; GSI, global severity index; IE, interpersonal effectiveness; PDSI, positive symptom distress index; PST, positive symptom total; RA, research assistant; RCTs, randomized controlled trials; SCID-I, structured clinical interview for DSM-IV, Axis I; SCID-II, structured clinical interview for DSM-IV, Axis II

## Footnotes

1 White and colleagues 1. White A, Jendritza T, Kim S, Homan J, Johnson J. Group and individual variables as predictors of graduation from a dialectical behavior therapy program. Paper presented at the annual meeting of the International Society for the Improvement and Teaching of Dialectical Behavior Therapy, Toronto, Canada. 2011.

2 Linehan DDS Linehan, M. M. (1982). Demographic Data Schedule (DDS). University of Washington, Seattle, WA, Unpublished work.
